# MAGE-C2在肺腺癌中的表达及临床意义

**DOI:** 10.3779/j.issn.1009-3419.2016.02.09

**Published:** 2016-02-20

**Authors:** 康 郭, 梦实 刘, 萍 徐, 红梅 李

**Affiliations:** 266003 青岛，青岛大学附属医院肿瘤中心 Cancer Center, the Affiliated Hospital of Qingdao Universitiy, Qingdao 266003, China

**Keywords:** 肺肿瘤, 肿瘤标志物, 黑色素抗原C2, Lung neoplasms, Tumor marker, MAGE-C2

## Abstract

**背景与目的:**

本研究旨在探讨黑色素抗原C2（melanoma antigen-C2, MAGE-C2）在肺腺癌组织及癌旁组织中的表达，及其在肺癌诊断及预后方面的临床指导意义。

**方法:**

利用荧光定量PCR和Western blot方法检测MAGE-C2 mRNA和MAGE-C2蛋白在87例肺腺癌患者的癌组织及癌旁组织中的表达。

**结果:**

肺腺癌组织中MAGE-C2 mRNA及MAGE-C2蛋白存在高表达（53/87, 60.9%），而癌旁组织中无MAGE-C2 mRNA和MAGE-C2蛋白的表达；统计学分析后发现，MAGE-C2的表达情况与年龄、性别、吸烟史无明显相关性（*P* > 0.05），而与肿瘤分期、是否转移及分化程度相关（*P* < 0.05）；MAGE-C2高表达对患者总生存率有不利的影响（*P* < 0.05）。

**结论:**

MAGE-C2在肺腺癌组织中存在高表达，并与肿瘤分期及是否转移存在相关性，有望成为一种新型的肺腺癌诊断、治疗及预后标志物。

## Abstract (en)

**Background and objective:**

The aim of this study is to detect the expression of melanoma antigen-C2 (MAGE-C2) in lung adenocarcinoma tissues and adjacent non-cancerousous tissues and analyze its clinical significance.

**Methods:**

The expression level of MAGE-C2 mRNA and MAGE-C2 protein were measured in 87 cases of lung adenocarcinoma tissues and adjacent non-cancerous tissues were detected by Real-time fluorescence quantitative PCR and Western blot.

**Results:**

Over-expression of the MAGE-C2 mRNA and MAGE-C2 protein were observed in lung adenocarcinoma tissues (53/87, 60.9%), but nor in adjacent non-cancerous tissues. The expressions of MAGE-C2 mRNA and MAGE-C2 protein were closely associated with clinical stage, metastasis and differentiation (*P* < 0.05). The expression rate of MAGE-C2 mRNA had no relationship with gender, age, smoking (*P* > 0.05). The expression of MAGE-C2 had an adverse effect on overall survival rate (*P* < 0.05).

**Conclusion:**

Over-expression of the MAGE-C2 mRNA and protein is existed in lung adenocarcinoma. The high expression of MAGE-C2 may be closely related to the occurrence and development in lung adenocarcinoma. MAGE-C2 may predict a poor prognosis and could be as a novel lung adenocarcinoma molecule marker.

肺癌是世界范围内最常见的肿瘤之一，而且其发病死亡率居首位^[1]^，是威胁人类生命健康的主要恶性肿瘤。其中肺腺癌的发病率逐年升高，已经跃居肺癌发病率首位^[2]^。研究^[2]^证实，早期诊断和治疗是提高肺癌患者生存率的关键。肿瘤标志物因其检测方便、成本低等优点可广泛用于肺癌的筛查，对肺癌早期诊断、组织学分型、预后判断及疗效评价等方面有重要价值^[3]^。肿瘤睾丸抗原（cancer-testis antigen, CTA）是一类新的肿瘤标志物，在多种肿瘤中存在表达，而在正常组织中仅表达于睾丸和胎盘组织^[4]^。黑色素抗原C2（melanoma antigen-C2, MAGE-C2）是CTA的一种，最初发现于黑色素瘤细胞，后被证实在多种肿瘤中存在表达^[5, 6]^，而其在肺腺癌中的研究却很少。本实验通过荧光定量PCR（realtime fluorescence quantitative PCR, RTFQ-PCR）及Western blot方法检测MAGE-C2 mRNA及蛋白在肺腺癌中的表达情况，并分析其与肺腺癌临床病理特征及总生存率之间的关系，以期望发现一种新的肺腺癌标志物。

## 1 资料与方法

### 1.1 病例资料

选取青岛大学附属医院胸外科2010年3月-2011年3月肺腺癌患者87例，分别取肺腺癌组织及癌旁组织10 g剪碎后储存于-80 ℃备用。癌旁组织取自癌组织旁5 cm以外的组织。所有患者均为接受过放化疗，并均已经病理诊断证实，且无其他恶性疾病及自身免疫性疾病。临床分期采用国际肿瘤-淋巴结-转移（tumor-node-metastasis, TNM）分期方法。

### 1.2 总RNA提取和cDNA合成

取冰冻肺腺癌组织及癌旁组织各50 mg-100 mg，利用RNAsimple Total RNA Kit提取总RNA，利用超微量核酸蛋白检测仪检测RNA纯度。再利用FastQuant RT Kit（with gDNase）进行cDNA合成。所有试剂盒均为天根科技有限公司提供，具体操作均按说明书进行。

### 1.3 荧光定量PCR检测

使用上述合成的cDNA为模板，利用SuperReal PreMix Plus（SYBR Green）试剂盒进行荧光定量PCR检测。所使用MAGE-C2上游引物为：5’-AAAGTCAGCACAGCAGAGGAG-3’，下游引物为：5’-TCTTCAGGAGCAGCAGGTAAA-3’。先95 ℃预变性15 min，再与荧光定量PCR仪（Bio-Rad仪器）内扩增40个循环（95 ℃变性10 s，55 ℃退火20 s，72 ℃延伸20 s），然后进行融解曲线分析。所有试剂盒均为天根科技有限公司提供，具体操作按说明书进行^[7]^。

### 1.4 Western blot检测

将冻存的组织标本取出称重，每100 mg加入500 μL蛋白裂解液，置于匀浆器中研磨，冰上裂解30 min，离心（12, 000 r/min）收集上清液，使用BCA法测定蛋白浓度。将蛋白于10%聚丙烯酰胺凝胶电泳分离，电转膜1.5 h，5%BSA封闭1 h后，分别加入MAGE-C2兔抗人单克隆抗体（1:1, 000）及GAPDH小鼠抗人单克隆抗体（1:1, 000），4 ℃孵育过夜漂洗，加二抗（1:2, 000）室温孵育1 h后加发光试剂，暗室曝光显示条带^[8]^。

### 1.5 随访

对87例患者进行长期随访，随访时间从诊断日期开始至最终观察日期或死亡日期。记录患者的总生存时间。

### 1.6 统计学方法

所有数据采用SPSS 19.0统计学软件进行分析。计量资料以Mean±SD表示，采用*t*检验；计数资料采用χ^2^检验；生存分析采用*Kaplan*-*Meier*法及*Cox*风险比例回归模型，以*P* < 0.05为差异有统计学意义。

## 2 结果

### 2.1 MAGE-C2 mRNA在肺腺癌及癌旁组织中的表达

超微量核酸蛋白检测仪检测RNA纯度，选取A260/A280的比值在1.8到2.1之间的样本用于合成cDNA。荧光定量PCR结果显示MAGE-C2 mRNA在肺腺癌组织存在表达（53/87, 60.9%），而在癌旁组织中无表达。

### 2.2 MAGE-C2蛋白在肺腺癌及癌旁组织中的表达

Western blot结果与荧光定量PCR检测结果一致，在肺腺癌组织中检测到MAGE-C2蛋白的表达（53/87, 60.9%）（图 1），而在癌旁组织中无表达。

**1:**
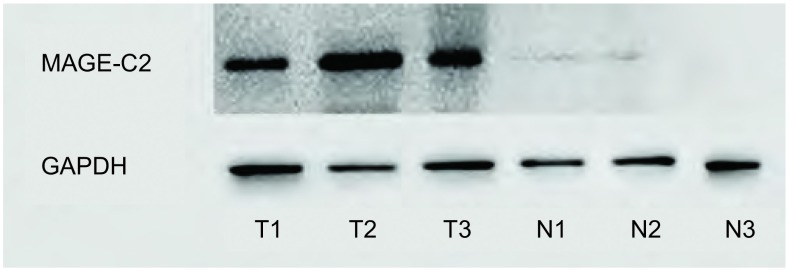
Western blot检测MACE-C2蛋白在肺腺癌及癌旁组织中的表达情况 Expression of MAGE-C2 protein was detected in lung adenocarcinoma and adjacent non-cancerous tissue by Western bolt. MAGE-C2: melanoma antigen-C2.

### 2.3 MAGE-C2 mRNA及蛋白表达与肺腺癌临床病例特征之间的关系

将MAGE-C2 mRNA及蛋白表达情况与肺腺癌患者的临床病理资料结合进行分析。我们发现，MAGE-C2 mRNA及蛋白表达阳性组与表达阴性组之间在临床分期、是否转移、分化程度方面存在统计学差异（*P* < 0.05）（表 1）；在MAGE-C2 mRNA表达阳性组中，晚期、低分化、转移组的表达量明显高于早期、高/中分化、无转移组（*P* < 0.05）（图 2）；但与患者的年龄、性别、吸烟史之间无明显相关性（*P* > 0.05）（表 1）。

**1:** MAGE-C2 mRNA及蛋白表达情况与临床病理特征的关系 Relationship between expression level of MAGE-C2 mRNA and protein and clinical characteristics of patients

Clinical characteristics	Cases (*n*)	MAGE-C2 mRNA and MAGE-C2 protein expression		*χ*^2^	*P*
		Positive	Negative		
All patients	87				
All tissues				47.306	< 0.01
Tumor	87	53	34		
ANCT	87	0	87		
Gender				0.940	> 0.05
Male	41	23	18		
Female	46	30	16		
Age (Year)				1.247	> 0.05
≥60	23	12	11		
< 60	64	41	23		
Smoking				0.917	> 0.05
Non-smoker	25	17	8		
Smoker	62	36	26		
TNM stage				5.455	< 0.05
Ⅰ+Ⅱ	52	27	25		
Ⅲ+Ⅳ	35	26	9		
Differentiation				5.744	< 0.05
Well/Mod	18	7	11		
Poor	69	46	23		
Metastasis				6.069	< 0.05
Negative	46	23	23		
Positive	41	30	11		
ANCT: adjacent non-cancerous tissue; TNM: tumor-node-metastasis.					

**2:**
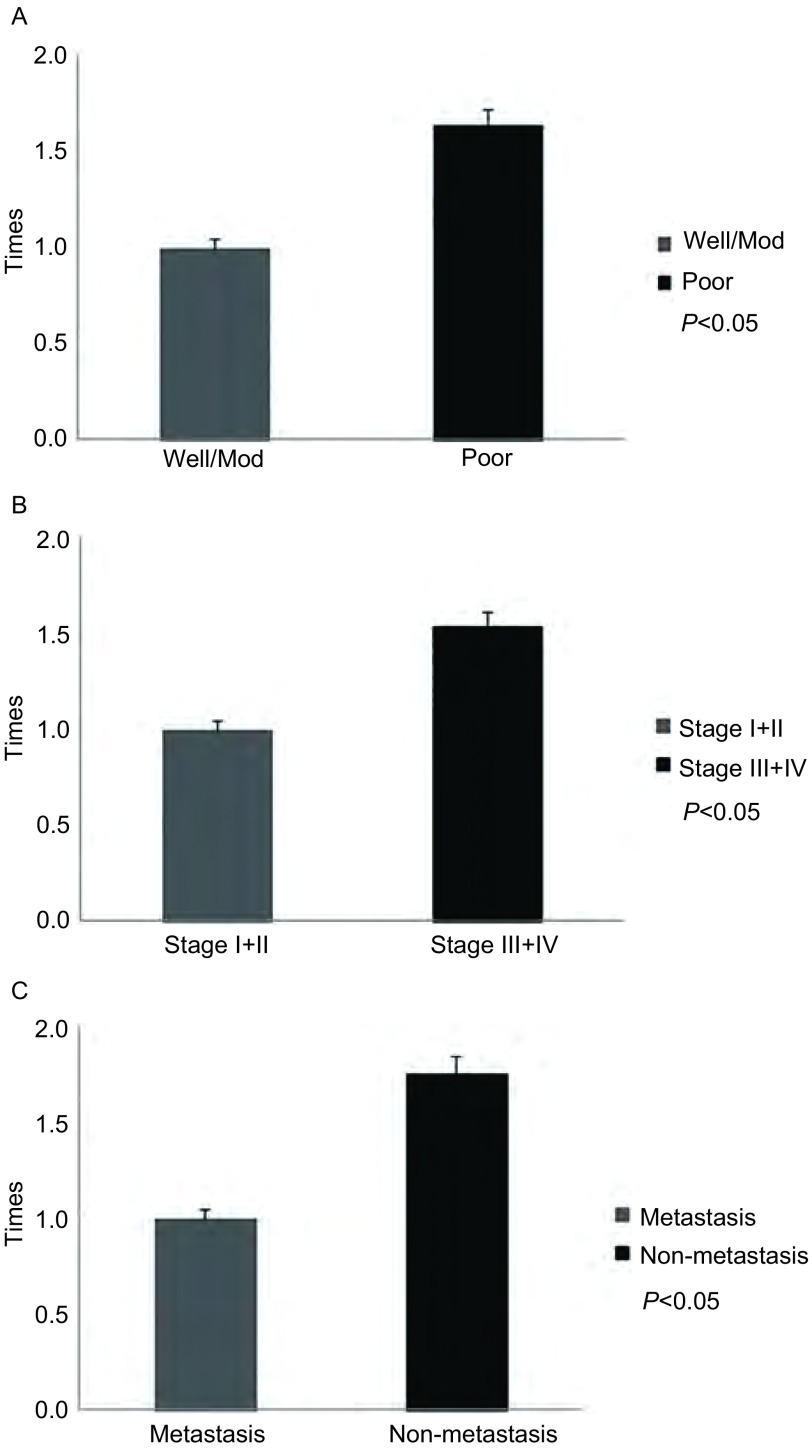
荧光定量PCR检测MAGE-C2 mRNA在肺腺癌中的表达情况。A：MAGE-C2 mRNA在高/中分化组和低分化组的表达情况（以高/中分化组为标准，低分组的荧光表达量）；B：MAGE-C2 mRNA在（Ⅰ期+Ⅱ期）组和（Ⅲ期+Ⅳ期）组的表达情况（以Ⅰ期+Ⅱ期组为标准，Ⅲ期+Ⅳ期组的荧光表达量）；C：MAGE-C2 mRNA在转移组和无转移组的表达情况（以无转移组为标准，转移组的荧光表达量）。 Expression of MAGE-C2 mRNA was detected in lung adenocarcinoma by realtime fluorescent quantitative PCR. A: MAGE-C2 mRNA expression in well/mod differentiation group and poor differentiation group (Compared to well/mod differentiation group, expression of MAGE-C2 mRNA in poor differentiation group); B: MAGE-C2 mRNA expression in stage Ⅰ+Ⅱ group and stage Ⅲ+Ⅳ group (Compared to stage Ⅰ+Ⅱ group, expression of MAGE-C2 mRNA in stage Ⅲ+Ⅳ group); C: MAGE-C2 mRNA expression in metastasis group and non-metastasis group (Compared to metastasis group, expression of MAGE-C2 mRNA in non-metastasis group).

### 2.4 MAGE-C2 mRNA与患者预后之间的关系

通过对87例随访数据进行*Kaplan*-*Meier*法生存分析，我们发现MAGE-C2 mRNA及蛋白表达阳性组与表达阴性组之间在总生存期上存在明显差异（*Log*-*rank*=7.541, *P* < 0.05）（图 3）。利用*Cox*风险比例回归模型对87例患者的7个因素进行分析，这7个因素分别为：年龄、性别、吸烟史、分化程度、临床分期、是否转移、MAGE-C2表达，结果发现，临床分期、是否转移、MAGE-C2表达分别为肺腺癌预后不良的独立因素。MAGE-C2 mRNA及蛋白高表达对患者总生存期产生不利影响（RR=2.813, 95%CI 1.714-4.616, *P*=0.017）（表 2），并可能与临床分期、是否转移间存在协同作用。

**3:**
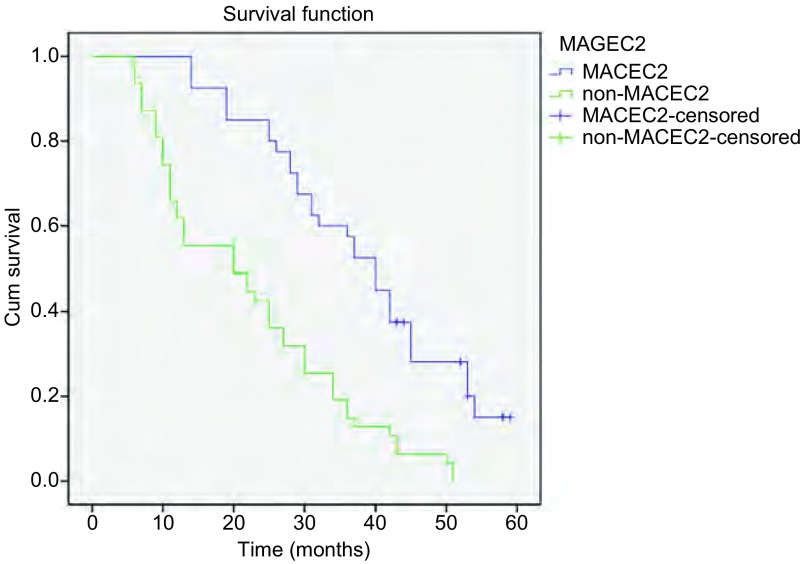
MAGE-C2 mRNA及蛋白的表达情况与肺腺癌患者总生存率之间的关系（*Kaplan*-*Meier*法） Relationship between expression of MAGE-C2 mRNA and protein and overall survival rate of lung adenocarcinoma patients (*Kaplan*-*Meier* method)

**2:** 肺腺癌预后因素的*Cox*风险回归模型分析 *Cox* multivariate analysis of prognostic factors of lung adenocarcinoma

Variables	RR	95%CI	*P* value
MAGE-C2	2.813	1.714-4.616	0.017
Gender	0.908	0.523-1.577	0.732
Age	0.989	0.967-1.012	0.359
Smoking history	1.780	0.983-3.222	0.057
Clinical stage	2.120	1.266-3.550	0.004
Differentiation	1.073	0.598-1.926	0.814
Metastasis	4.014	2.416-8.016	< 0.000, 1

## 3 讨论

CTA抗原基因编码的肿瘤抗原能在多种肿瘤组织中表达，但在正常组织中仅限于睾丸和胎盘组织，但是睾丸不表达人类白细胞抗原（human leukocyte antigen, HLA），因此不会在体内产生相应抗体^[9, 10]^。Jassim等^[11]^的研究发现CTA的异位表达能引发人体的免疫反应，在血液中产生相应抗体，这为CTA在早期肿瘤诊断方面的应用提供了依据^[12]^。另外，由于CTA这种免疫原性及表达限制性，CTA在肿瘤的诊断及免疫治疗方面存在巨大的潜在价值。

MAGE-C2是CTA家族中的一员，可在肿瘤细胞的胞质内被降解成九肽或十肽，然后作为抗原表位，与细胞内的HLA分子结合后呈递到细胞膜^[13]^，从而诱导机体免疫系统产生相应抗体。在本研究中，我们检测了87例肺腺癌患者癌组织及癌旁组织中MAGE-C2 mRNA及蛋白的表达情况，结果发现，MAGE-C2 mRNA及蛋白表达于多数肺腺癌组织中（53/87, 60.9%），而在癌旁组织中未检测到MAGE-C2 mRNA及蛋白的表达。因此，MAGE-C2有潜力作为一种新型的肺腺癌标志物，其在血清中产生的特异性抗体可广泛用于肺癌的筛查。另外，通过分析MAGE-C2 mRNA及蛋白的表达情况与肺腺癌临床病理特征之间的关系，我们发现，MAGE-C2 mRNA及蛋白的表达阳性组与表达阴性组在肺腺癌的临床分期、是否转移及分化程度方面存在明显统计学差异（*P* < 0.05），并且在晚期、低分化、转移组的表达量明显高于早期、高/中分化、无转移组。这一结果提示，MAGE-C2的高表达可能对肿瘤细胞的分化、转移有促进作用，其具体机制有待进一步研究。

利用*Cox*风险比例回归模型及*Kaplan*-*Meier*法对随访数据进行生存分析后，我们发现，MAGE-C2的高表达同临床分期、是否转移一样，为肺腺癌不良预后的独立因素。MAGE-C2表达越高，预后相对越差。因此，我们认为，MAGE-C2不仅可以用于肺腺癌的诊断，而且可以作为肺腺癌不良预后的指标。另外，由于其特殊的免疫原性及表达限制性，MAGE-C2可能作为肺腺癌免疫治疗的靶点。体外培养能结合MAGE-C2的细胞毒性T淋巴细胞，进而特异性杀伤体内肿瘤细胞，而不对正常组织造成损害，减少了肿瘤治疗的副反应^[14]^。同时，阻断MAGE-C2相关的信号转导途径^[15]^，可能达到延缓肿瘤转移、改善患者的预后目的。相信此方面的研究将会对肿瘤的临床治疗产生重大影响。

总体来看，本研究成功证实了MAGE-C2在肺腺癌组织中存在高表达，并与肺腺癌的临床分期、是否转移及分化程度相关，对患者的总生存期有不良影响。这表明，MAGE-C2可作为一种肺腺癌诊断及预后标志物，并可作为免疫治疗靶点，在临床应用中存在巨大潜力。
